# Hikikomori Is Most Associated With Interpersonal Relationships, Followed by Suicide Risks: A Secondary Analysis of a National Cross-Sectional Study

**DOI:** 10.3389/fpsyt.2019.00247

**Published:** 2019-04-16

**Authors:** Roseline Yong, Kyoko Nomura

**Affiliations:** Public Health Department, The Graduate School of Medicine, Akita University, Akita, Japan

**Keywords:** hikikomori, social withdrawal, interpersonal difficulties in hikikomori, suicide risk factors in hikikomori, anxieties in hikikomori, Japan, psychiatric treatment

## Abstract

There have been few population studies of *hikikomori* (that is, prolonged social withdrawal and isolation), and the basic correlating factors of hikikomori are yet to be identified. Therefore, this study aimed to identify the associated basic characteristics and psychiatric factors of hikikomori. Data were obtained from the Survey of Young People’s Attitudes of 5,000 residents (aged 15–39 years) who were randomly selected from 200 urban and suburban municipalities in Japan in February 2010. The chi-square test and multiple logistic regression were used in the analysis. The data contained 3,262 participants (response rate: 65.4%); 47.7% were men (*n* = 1,555) and 52.3% were women (*n* = 1,707). Its prevalence was 1.8% (*n* = 58), and 41% had been in the hikikomori state for more than 3 years. There were fewer hikikomori people in neighborhoods filled with business and service industries. Significantly more men were in the hikikomori group (65.5%) than in the non-hikikomori group (47.3%). The hikikomori group was more likely to drop out of education (*p* < .001) and to have a psychiatric treatment history compared with non-hikikomori (37.9% vs 5%, *p* < .001). The multiple logistic regression analyses revealed that interpersonal relationships were significantly associated with hikikomori across three models (Model 1 adjusting for all basic characteristics, OR = 2.30, 95% CI = 1.92–2.76; Model 2 further adjusting for mental health-related factors, OR = 2.1, 95% CI = 1.64–2.68; Model 3 further adjusting for a previous psychiatric treatment history, OR = 1.95, 95% CI = 1.52–2.51). Additionally, the hikikomori group was more likely to have suicide risk factors (Model 1: OR = 1.85, 95% CI = 1.56–2.20; Model 2: OR = 1.33, 95% CI = 1.05–1.67), obsessive–compulsive behaviors (Model 1: OR = 1.57, 95% CI = 1.20–2.05), and addictive behaviors (Model 1: OR = 1.93, 95% CI = 1.37–2.70). This is the first study to show that hikikomori is associated with interpersonal relationships, followed by suicide risks. Hikikomori people are more likely to be male, have a history of dropping out from education, and have a previous psychiatric treatment history.

## Introduction

The term *hikikomori* refers to a social condition in which people avoid social participation and having relationships with people besides family members by confining themselves to a room or the house for 6 months and more. The term refers to both the condition itself and the people who suffer from it. Although the phenomenon is thought to be distinguishable from mental illness, new guidelines have warned that mental health problems, such as schizophrenia, may have been underdiagnosed (1).

There have been few epidemiological studies of hikikomori that use community samples. In Japan, there have been three national surveys of hikikomori among the general population. The first was a nationwide cross-sectional mental health study in 2002–2006 that estimated that 0.56% of all households had at least one ongoing hikikomori case. The same study also reported that 1.2% of the interviewees had a lifetime prevalence of hikikomori (age 20–49 years, response rate: 55.1%, *n* = 4,134), and that 54.5% of them had also experienced a psychiatric disorder (mood, anxiety, impulse control, or substance-related) in their lifetime (2). The second and third surveys, which were the Survey of Young People’s Attitudes (Fact-finding Survey on Social Withdrawal) (SYPA) conducted by the Cabinet Office of Japan, revealed that the prevalence of hikikomori among people aged 15–39 years was 1.79% in 2009 (response rate: 65.7%, *n* = 3,287) and 1.57% in 2015 (response rate: 62.3%, *n* = 3,115). Among the hikikomori people, approximately 67% were reported to be unemployed. In both Cabinet Office surveys, people with schizophrenia, who were pregnant, or who were a homemaker and who shared the hikikomori definition of staying at home for 6 months or longer due to family responsibilities were not counted as hikikomori (3, 4).

Although hikikomori was once thought to be a culture-bound syndrome unique to Japan (5), cases have subsequently been reported in Oman (6), Spain (7–9), South Korea (10,), Canada (12, 13), Hong Kong (14–16), India (11), France (17), Austria (18), China (18, 19), the United States (11), and Brazil (20). Aside from these case reports, surveys of psychiatrists from countries as diverse as Australia, Bangladesh, Iran, Taiwan, and Thailand suggest that hikikomori cases have been observed and examined in all these countries, and that psychological factors are common causes of hikikomori (21). The same study also shows that various diagnoses were given, indicating that many of the psychiatrists believe that hikikomori is an outcome behavior of a given disorder that requires treatment.

In fact, in Japan, almost half of the limited cases presented to health centers get diagnosed. Among these, one-third of the subjects are diagnosed with schizophrenia, mood disorders, or anxiety disorders, suggesting that pharmacotherapy is needed. Others are diagnosed with personality disorders or pervasive developmental disorders, indicating that psycho-social support is more appropriate (22). Half of the people with lifetime hikikomori were found to have a comorbid mood disorder (2). Some consider that hikikomori is a result of conflicting demands and the reduced autonomy of the individual (18), which is trigged by stressful events and combined with a predisposed introverted personality (12). Others think that the hikikomori phenomenon may be a preferred lifestyle among the younger generations (14) and that it is more common in urban areas (21). Despite the ambiguous findings about hikikomori that have mainly been collected from the specialists’ opinions and psychiatric referrals, the hikikomori phenomenon has greatly affected the health, labor power, and welfare of Japan as the youth unemployment rate has been a concern since the 1990s (23). Therefore, it is important to identify the associated sociodemographic and psychiatric factors of being a hikikomori.

As there have been few epidemiological studies of hikikomori, many of its factors remain unknown. Thus, population studies are necessary to identify the basic characteristics of hikikomori as well as its correlations with general mental health risk factors. To fill this gap, we conducted a secondary analysis using the SYPA data (3) to identify the factors associated with hikikomori. The SYPA data are well-designed, randomized, and contain much valuable information about the sociodemographic and psychiatric factors.

## Methods

This study was approved by the ethics committee of Akita University Graduate School of Medicine. The SYPA 2010 data (3) were obtained from the Social Science Japan Data Archive, and the variables were re-categorized for secondary analysis. As the data are not individually identifiable, the written informed consent of the participants was not required.

### Sampling

**Figure 1:**
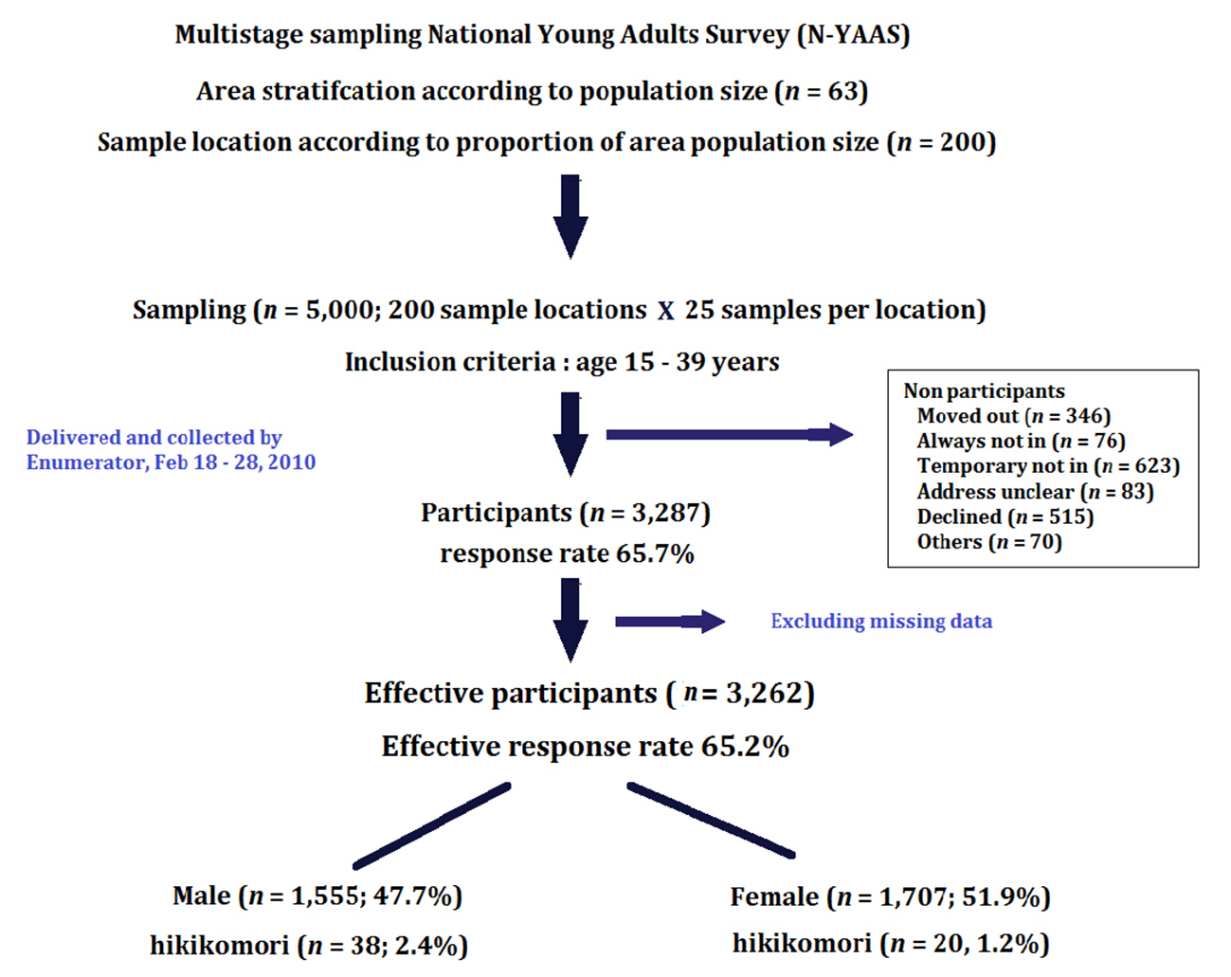
Flow chart of the sampling procedure.

A total sample size of 5,000 was estimated for the population of 15–39-year-olds. Multistage stratified randomized sampling was used to ensure that the samples represented all areas in Japan. Firstly, 200 locations were randomly selected from 198 municipalities stratified by area and population size. Secondly, in each location, 25 samples were randomly selected from the municipality registration list. A set of self-administered questionnaires was distributed and collected by hand between February 18 and February 28, 2010. The response rate was high (65.7%): 3,287 participants responded to the study and, after excluding missing data, 3,262 samples were effective for analysis (**Figure 1**).

### Outcome Variable

The outcome variable was hikikomori, for which there were three major screening questions. Firstly, the participants were asked to choose one item from the following multiple-choice question about the frequency of going out: “How frequently do you leave your house?” The multiple-choice answers were 1) “I go out every day for work or school”; 2) “I go out 2–4 days per week for work or school”; 3) “I go out frequently for fun and so on”; 4) “I sometimes go out to mingle with others”; 5) “I stay at home most of the time, and I only go out when there is something that interests me”; 6) “I stay at home most of the time, but I may go out to the convenience stores nearby”; 7)” I do leave my room but do not go out of the house”; and 8) “I stay only in my room.”

Those who selected options 5–8 then proceeded to the next question that asked about the duration of their behaviors. Those who gave a duration of 6 months and above were classified as belonging to the hikikomori group. Then, they were screened for the exclusion criteria in the third question that asked for the reasons for their social disengagement. Those who declared that the reason for staying mainly at home was because of pregnancy, doing housework, being a homemaker, or being diagnosed with schizophrenia were excluded from the classification.

### Exposure Variables

The exposure variables included personal demographics and psychiatric factors. The personal demographics included sex, age, city size, region, number of household members, social class, neighborhood characteristics (the housing area, shops and service industries, factories, agriculture/forestry/fishery, if people lived there for many years, close neighborhoods, rich social activities, rich local events, and others), and educational status.

We measured psychiatric factors with 20 simple yes/no questions (**Table 2**). These items were further grouped into five different psychiatric factors [suicide risks, violent tendencies, interpersonal difficulties, obsessive–compulsive behaviors (OCBs), and dependency behaviors] to assess the risks of the different groups. A “yes” for a single item counted as 1 point. The items assessing suicide risks (0–5 points) were as follows: “I often feel guilty towards family,” “I often feel that my life is suffocated,” “I wish to die,” “I always feel hopelessness,” and “I hurt myself (e.g., cut my wrist).” The items assessing violent tendencies (0–4 points) were as follows: “I hit my family members,” “I hit the walls or windows,” “I throw and destroy things occasionally (e.g., dishes),” and “I occasionally yell at others.” The items assessing interpersonal difficulties (0–4 points) were as follows: “I am afraid of meeting others,” “I am anxious about the possibility of meeting people that I know,” “I am anxious about what others might think of me,” and “I cannot blend into groups.” The items assessing OCB (0–4 points) were as follows: “I cannot stand it if meal and bath times are slightly different than usual,” “I pay excessive attention to my own cleanliness,” “I excessively double-check things and have repetitive thoughts,” and “I repeat the same act over and over.” Finally, dependency behaviors were assessed with the following items (0–3 points): “I cannot stop drinking,” “I depend heavily on medications,” and “I am anxious if I am away from my phone or computer for even a moment.”

### Statistical Analysis

The basic characteristics and variables of interest were compared between the groups with and without hikikomori using the chi-square test of independence (with the Yate’s continuity correction). Effect sizes were calculated using phi coefficient (small = .10, medium = .30, large = .50) and Cramer’s V (small = .06, medium = .17, large = .29) (24). *Post hoc* analysis was performed to determine the association between hikikomori and the exact individual items. Considering the possibility of the multiple comparisons problem of the multi-item test for the psychiatric factors, significance levels were adjusted for the number of items. Logistic regression was performed to identify the factors associated with being hikikomori, and the odds ratios were estimated along with the 95% confidence intervals (95% CI). Three models were employed in the multiple logistic regression analysis: Model 1 was adjusted for all the basic characteristics, Model 2 was further adjusted for all the tested psychiatric factors, and Model 3 was adjusted for the history of psychiatric treatment in addition to the factors tested in Model 2. All analyses were performed using SPSS v. 17.0 (SPSS Inc., Chicago, IL, USA), and the significance level was *p* < .05.

## Results

The data contained 3,262 participants (effective response rate: 65.4%) of whom 47.7% were men (*n* = 1,555) and 52.3% were women (*n* = 1,707). The prevalence of hikikomori was 1.8% (*n* = 58: men *n* = 38, women *n* = 20). Among them, 41% had been in a hikikomori state for more than 3 years. There were significantly fewer people in the hikikomori group living in an area that is filled with business and service opportunities (3.4% vs 13.3%, *p* = .045, *phi* = −.039), whereas the numbers according to the city size, region, number of family members, and social class were not significantly different. The chi-square test showed that there were significantly more men in the hikikomori group than in the non-hikikomori group (65.5% vs 47.3%, *p* < .001, *phi* = .05), and significantly more hikikomori had dropped out of the education system (19% vs 3.2%, *p* < .001, *Cramer’s V* = .195). A further analysis was performed to determine the exact differences between the participants of different educational statuses; because of the small numbers in each group, people who dropped out and were taking time off were combined for analysis, and people who had not answered were excluded. This *post hoc* analysis showed that the people who had dropped out or were taking time off from their studies were mostly in the hikikomori group rather than in the non-hikikomori group (standard residuals = 8.2). Significantly more hikikomori had a previous history of psychiatric treatment (37.9% vs 5%, *p* < .001, *phi* = .19; **Table 1**). In **Table 2**, the chi-square test showed that there were significantly more hikikomori than non-hikikomori who had one or more suicide risk factors (81.0% vs 43.6%, *p* < .001, *phi* < .001), one or more interpersonal difficulties (74.1% vs 36.0%, *p* < .001, *phi* < .001), one or more OCBs (39.7% vs 24.0%, *p* = .006, *phi* = .006), and one or more dependency behaviors (25.9% vs 15.0%, *p* = .022, *phi* = .022). The *post hoc* analysis revealed that the chi-square test results also showed that significantly more people in the hikikomori group had suicide risk factors (all *p* < .001,.073 ≤ *phi* ≤.111) and interpersonal difficulties (all *p* < .001,.069 ≤ *phi* ≤.203), but an association was only partially observed in those with OCBs and violent tendencies. Furthermore, significantly more hikikomori people had a dependency on medications (12.1% vs 1.9%, *p* < .001, *phi* = .094).

**Table 1:** Basic characteristics of the participants (*N* = 3,262).

		Hikikomori (*n* = 58)	Non-hikikomori (*n* = 3,204)	*p*-value
Sex	Male	38 (65.5%)	1,517 (47.3%)	.009^b^
Age	15–19 years old 20–24 years old 25–29 years old 30–34 years old 35–39 years old	9 (15.5%) 12 (20.7%) 11 (19%) 13 (22.4%) 13 (22.4%)	588 (18.4%) 498 (15.5%) 584 (18.2%) 687 (21.4%) 847 (26.4%)	.808^a^
City size	Metropolitan cities Medium cities (population ≥ 200,000) Smaller cities (population ≥ 100,000) Town and villages (population < 100,000)	13 (22.4%) 12 (20.7%) 15 (25.9%) 18 (31.0%)	811 (25.3%) 864 (27.0%) 526 (16.4%) 1,003 (31.3%)	.252^a^
Area	Hokkaido Tohoku Kanto Chubu Kinki Chugoku/Shikoku Kyushu	6 (10.3%) 7 (12.1%) 19 (32.8%) 8 (13.8%) 8 (13.8%) 5 (8.6%) 5 (8.6%)	115 (3.6%) 223 (7.0%) 1,056 (33.0%) 644 (20.1%) 486 (15.2%) 275 (8.6%) 405 (8.6%)	.087^a^
Neighborhood characteristics	Residential housing area Shops and service industries Factories Agriculture/forestry/fishery Residents have lived there for many years Close neighborhood Rich social activities Rich local events None of the above No response	39 (67.2%) 2 (3.4%) 1 (1.7%) 7 (12.1%) 31 (53.4%) 15 (25.9%) 5 (8.6%) 9 (15.5%) 2 (3.4%) 0	2,256 (70.4%) 426 (13.3%) 138 (4.3%) 207 (6.5%) 1,649 (51.5%) 670 (20.9%) 445 (13.9%) 679 (21.2%) 85 (2.7%) 3 (0.1%)	.705^b^ .045^b^ .524^b^ .149^b^ .868^b^ .450^b^ .337^b^ .375^b^ >.999^b^ >.999^b^
Education status	Currently studying Finished studying Dropout Time off No answer	8 (13.8%) 36 (62.1%) 11 (19.0%) 3 (5.2%) 0	733 (22.9%) 2,355 (73.5%) 102 (3.2%) 3 (0.1%) 11 (0.3%)	<.001^a^
Number of family members	Staying alone Staying with others (2–4 people) Staying with others (5 people and above)	5 (8.6%) 43 (74.1%) 10 (17.2%)	171 (5.3%) 2,172 (67.8%) 861 (26.9%)	.178^a^
Social class	Upper class Middle class Lower class	2 (3.4%) 45 (77.6%) 11 (19%)	147 (4.6%) 2,589 (80.8%) 468 (14.6%)	.615^a^
History of psychiatric treatment	Yes	22 (37.9%)	160 (5%)	<.001^b^

a p-value derived using the Pearson’s chi-square test.

b p-value derived using the continuity correction computer only for a 2 × 2 table chi-square test.

**Table 2:** Psychiatric factors of the participants (*N* = 3,262).

	Hikikomori (*n* = 58)	Non-hikikomori (*n* = 3,204)	*p*-value
Suicide risks (one risk or more) I often feel guilty toward my family I often feel that my life is suffocated I wish to die I always feel hopeless I hurt myself (e.g., cut my wrist)	47 (81.0%) 42 (72.4%) 28 (48.3%) 21 (36.2%) 19 (32.8%) 5 (8.6%)	1,397 (43.6%) 1,046 (32.6%) 649 (20.3%) 340 (10.6%) 438 (13.7%) 32 (1.0%)	<.001 <.001* <.001* <.001* <.001* <.001*
Violent tendencies (one risk or more) I hit my family members I hit the walls or windows I throw and destroy things occasionally (e.g., dishes) I occasionally yell at others	10 (17.2%) 3 (5.2%) 6 (10.3%) 5 (8.6%) 5 (8.6%)	470 (14.7%) 81 (2.5%) 224 (7.0%) 35 (1.1%) 329 (10.3%)	.584 .4 .465 <.001* .848
Interpersonal difficulties (one risk or more) I am afraid of meeting others I am anxious about the possibility of meeting people that I know I am anxious about what others might think of me I cannot blend into groups	43 (74.1%) 21 (36.2%) 28 (48.3%) 30 (51.7%) 31 (53.4%)	1,155 (36.0%) 260 (8.1%) 227 (7.1%) 906 (28.3%) 467 (14.6%)	<.001 <.001* <.001* <.001* <.001*
OCB (one risk or more) I cannot stand it if my meals and bath times are slightly different than usual I pay excessive attention to my own cleanliness I excessively double-check things and have repetitive thoughts I repeat the same act over and over	23 (39.7%) 1 (1.7%) 8 (13.8%) 17 (29.3%) 14 (24.1%)	769 (24.0%) 45 (1.4%) 236 (7.4%) 493 (15.4%) 324 (10.1%)	.006 >.999 .111 .007* .001*
Dependencies (one risk or more) I cannot stop drinking I am heavily dependent on medications I am anxious if I am away from my phone or computer for even a moment	15 (25.9%) 5 (8.6%) 7 (12.1%) 9 (15.5%)	479 (15.0%) 204 (6.4%) 61 (1.9%) 291 (9.1%)	.022 .671 <.001* .147

p-value derived using the continuity correction computer only for a 2 × 2 table chi-square test.

* = p-values that meet the significance level after being adjusted for the number of items. OCB, obsessive–compulsive behavior.

The multiple logistic regression analyses using the psychiatric factors as continuous variables revealed that interpersonal relationships were consistently and significantly associated with being hikikomori across the three models (Model 1, OR = 2.30, 95% CI: 1.92–2.76; Model 2, OR = 2.1, 95% CI: 1.64–2.68; Model 3, OR = 1.95, 95% CI: 1.52–2.51; **Table 3**). In addition, Model 1 revealed that the hikikomori group was more likely to have more suicide risks (OR = 1.85, 95% CI: 1.56–2.20), more OCBs (OR = 1.57, 95% CI: 1.20–2.05), and more dependency behaviors (OR = 1.93, 95% CI: 1.37–2.70). In Model 2, only suicide risk factors (OR = 1.33, 95% CI = 1.05–1.67) remained significant. The significance of suicide risks was no longer observed in Model 3. Among the baseline characteristics that were input into the multiple logistic models, only sex was significantly associated with being hikikomori. Males were more likely to become hikikomori (*p* < .01 in Model 1 and Model 2, *p* < .001 in Model 3). In addition, a history of psychiatric treatment was significantly associated with being hikikomori (*p* < .001 in Model 3). The multiple logistic regression analyses results of one or more of the different psychiatric factors are shown in **Supplementary Table 1**. The results are consistent with the results in **Table 3** in terms of the direction of the association and significance.

**Table 3:** Association between the hikikomori condition and psychiatric factors.

	Model 1 OR (95% CI)	Model 2 OR (95% CI)	Model 3 OR (95% CI)
Suicide risks	1.85 (1.56–2.20)	1.33 (1.05–1.67)	1.24 (0.98–1.57)
Violent tendencies	1.29 (0.91–1.83)	0.87 (0.59–1.28)	0.95 (0.64–1.41)
Interpersonal difficulties	2.30 (1.92–2.76)	2.10 (1.64–2.68)	1.95 (1.52–2.51)
OCB	1.57 (1.20–2.05)	0.78 (0.55–1.09)	0.80 (0.56–1.14)
Dependencies	1.93 (1.37–2.70)	1.16 (0.79–1.72)	0.96 (0.64–1.45)

Model 1 = Odds ratio (OR) adjusted for age, sex, number of family members, and social class.

Model 2 = Odds ratio adjusted for age, sex, numbers of family members, social class, and all psychiatric factors.

Model 3 = Odds ratio adjusted for age, sex, numbers of family members, social class, all psychiatric factors, and history of psychiatric treatment.

## Discussion

This is the first study to show that being hikikomori is closely associated with interpersonal relationships followed by suicide risks. Hikikomori are more likely to be male, have dropped out from school, and have a history of previous psychiatric treatment. In addition, Japanese hikikomori are less likely to reside in a neighborhood filled with business and service industries.

### Influence of Psychiatric Factors on Hikikomori

#### Interpersonal Difficulties Expressed as Anxieties

Our results showed that interpersonal difficulties were the most significant and strongest indicator for hikikomori. The items related to interpersonal difficulties included questions about anxiety toward specific objects (namely, people that the person knows). One item, “I cannot blend into a group,” implies that hikikomori have difficulties blending in with others and fitting into a group. This particular difficulty may be governed by a lack of communication skills or consequent feelings of alienation if their communication skills are not the problem. Another item, “I am anxious about the possibility of meeting people that I know,” indicates that the fear of familiar people is a unique characteristic of hikikomori. Combined with two other items, “I am afraid of meeting others” and “I am anxious about what others might think about me,” it appears that the fear of not meeting expectations may govern these anxieties. These anxieties may be related to a sense of humiliation, which suggests that they are afraid of being seen in their current situation. This echoes the findings of previous studies that have identified that anxieties in hikikomori may be related to poor self-identity that developed during early adolescence (1, 18). Unlike anxieties found in social phobias or generalized social anxieties (25), in which the fear is of a wide range of objects (and not specific objects), our finding of an association between hikikomori and interpersonal difficulties indicates that hikikomori fear people and the community that they know. By carefully assessing the types of fears that they may have, our data suggest the possibility that improving communication skills and managing expectations may be helpful for combating hikikomori. In fact, encouraging their sense of belonging to the community and helping them to reason with their fears have been shown to be effective for improving communication skills among the hikikomori, thus leading to recovery (26).

#### Higher Suicide Risk May Be Confounded by a Previous History of Psychiatric Treatment

Our study shows that people with one or more suicide risk factors have 2.8 times higher chances of being a hikikomori. Moreover, with the number of suicide risks, the risk of becoming hikikomori significantly increases. However, the difference was not significant after controlling for previous history of psychiatric treatment, suggesting that the suicide risks in the hikikomori are related to other factors associated with the previous history of psychiatric treatment, or to the effect of an existing psychiatric disorder other than the OCBs, violence, and addiction. However, we cannot ignore the suicide risk among the hikikomori, and it should be noted not only that suicide is the leading cause of death among people aged 20–39 years in Japan but also that almost one-third of suicides occur in the undefined unemployed group, which may indicate hikikomori (27). In addition, the previous literature reports that hikikomori have low self-worth that often leads to suicidal thoughts (28); thus, the condition of hikikomori requires active intervention (15, 16, 29) instead of the passive attitude stating that it is merely a lifestyle choice (14).

#### Other Significant Factors Associated With Being Hikikomori

The only significant difference between the hikikomori and non-hikikomori groups regarding violent tendencies was occasionally throwing and destroying things, such as dishes, although the numbers were low. This suggests that the expression of violence is more inward. Moreover, in our study, a larger proportion of hikikomori had self-harming behavior, which is further evidence of violence toward the self. An association between hikikomori and OCBs was observed in responses to the items “repeatedly checking on meaningless things or thoughts” and “repeating the same act over and over,” yet this influence was not observed after adjusting for other mental health indicators. Thus, OCBs are weakly associated with being hikikomori.

#### Psychiatric Treatment: Harmful or Beneficial?

In this study, 37.9% of the hikikomori had a previous history of psychiatric treatment, which suggests that mental health comorbidities are prevalent in the hikikomori. The higher proportion of hikikomori who are dependent on medication is also alarming. These findings indicate that psychiatric treatment does not guarantee social participation. We were unable to clarify if such dependency on medication is driven by existing psychiatric disorders, but we also cannot ignore the fact that the hikikomori symptoms may be related to the psychological factors associated with the treatment process, communication, and use of prescribed medications. Our data raise the simple question of “can psychiatric treatment elevate the hikikomori symptoms?” In the treatment guidelines for hikikomori, physicians are advised to carefully consider possible psychiatric diagnostic options (1), and in view of the fact that there is no evidence for whether psychiatric treatment promotes or prevents hikikomori, we suggest that a psychiatric treatment plan should be considered more carefully.

### Other Characteristics of Hikikomori

#### Are There More Men Than Women Who Are Hikikomori?

Our study provides the first epidemiological evidence of sex differences in hikikomori, which echoes the mainstream idea that there are more hikikomori men than women (5). In contrast, Koyama et al. did not find a significant difference between men and women who had identified themselves as having a lifetime prevalence of hikikomori (2). However, as the sample in Koyama’s study (2010) was people who had recovered from hikikomori, it does suggest that women tend to recover from the hikikomori situation better than men. By contrast, Yong et al. found no significant differences between the prevalence of hikikomori in men and women in rural areas (30). The current evidence is still limited regarding whether there is a gender difference in becoming hikikomori. More studies need to be conducted, and their results should be interpreted with extra caution, considering the characteristics of the samples.

#### More Dropouts

Our study provides the first epidemiological evidence for the influence of educational status on hikikomori. High school and university students who have dropped out from the education system may have a higher chance of becoming hikikomori. The positive adjusted residual value also further confirms that people who drop out or take time off from their studies are significantly more likely to be hikikomori than those who graduate or continue their studies. There are various reasons for dropping out, which we failed to explore in detail in this study. Financial difficulties, academic difficulties, sickness, and maladjustment have been found to be the major reasons for dropping out of university (31). It has been found that maladjustment in students is related to difficulties in the transition from high school to university (32, 33), that moving to a new city and away from the family environment is stressful (34), and that the varying and superficial relationships promote loneliness (35). Early prevention, such as providing advice, information, financial support, or companionship during the first year of college, may be helpful (34).

#### Possible Influence of the Residential Characteristics

Our study does not support the idea that hikikomori is more common in urban areas (21), as no association between the city size, region, and hikikomori was identified. Instead, hikikomori was found to be less common in residential areas that have many business and service industries. As these residential areas may contain diverse people and cultures, and more outdoor options and job opportunities, future studies should clarify if these factors are associated with hikikomori.

### Limitations and Strengths

There were several limitations in this study. Firstly, as self-reporting was used in this study, a misclassification bias may exist. We are also unsure if schizophrenia was really excluded from the classification of hikikomori. Secondly, we had no proper documentation of other psychotic disorders or data for depression. The simple yes/no response pattern to questions about mental health-related behaviors may not be a sufficient evaluation. Thirdly, psychological behaviors are often influenced by social events in the daily lives of individuals. However, as this Cabinet Office study focused more on the prevalence of hikikomori, it did not include questions about social and life events that might have influenced mental health-related behaviors. Another disadvantage of the secondary analysis of an existing dataset is that we lacked the variables of interest that we wished to study in more depth. Yet, there were also many advantages of using the existing data set. The SYPA is a large-scale population-based survey that would be difficult to conduct at an individual level. The identification of the variables, such as city size and region, is well-preserved and well-documented, which allowed us to examine the factors associated with hikikomori at different levels. The data collection process was also well-documented, which enabled us to consider more details during the analytic process.

## Conclusions

Our study is one of the very few population studies that have aimed to identify the social and health characteristics associated with being hikikomori. At a glance, people with hikikomori symptoms can also have other psychiatric symptoms, such as a suicide risk, OCBs, and addictive tendencies, and many of these psychiatric symptoms can be explained by interpersonal difficulties and a previous history of psychiatric treatment, if we are willing to make a closer examination. In contrast to some specialists’ opinions, hikikomori is not more common in urban areas than in rural areas. Being a man, having a history of dropping out from the educational system, and having a history of psychiatric treatment are contributing factors for hikikomori. By contrast, living in residential areas with many business and service industries can be a protective factor for hikikomori. Future studies should seek to verify the consistency of these findings, possibly using a cohort design.

## Ethics Statement

This study was approved by the ethics committee of Akita University Graduate School of Medicine.

## Author Contributions

RY contributed to the conception and design of the study, organized the database, performed the statistical analysis, and wrote the first draft of the manuscript. KN edited sections of the manuscript. All authors contributed to the manuscript revision and read and approved the submitted version.

## Funding

This study is funded by the Japan Society for the Promotion of Science, grant number 17K09191.

## Conflicts of Interest Statement

The authors declare that the research was conducted in the absence of any commercial or financial relationships that could be construed as a potential conflict of interest.
