# Supplementary material for: Hikikomori Is Most Associated With Interpersonal Relationships, Followed by Suicide Risks: A Secondary Analysis of a National Cross-Sectional Study
Source: Front Psychiatry. 2019 Apr 16;10:247. doi: 10.3389/fpsyt.2019.00247 (PMC6476969; doi:10.3389/fpsyt.2019.00247)
Supplement: Supplementary file 1 [file Table_1.pdf]

Supplementary Table 1: Sensitivity analyses of the association between the hikikomori condition and psychiatric factors (one or more risks) with psychiatric factors categorized into binary variables

	Model 1<br>OR (95% CI)	Model 2<br>OR (95% CI)	Model 3<br>OR (95% CI)
Suicide risks	5.36 (2.76-10.43)	3.22 (1.56-6.64)	2.83 (1.35-5.92)
Violent tendencies	1.23 (0.61-2.47)	0.74 (0.36-1.51)	0.71 (0.34-1.47)
Interpersonal difficulties	5.59 (3.06-10.19)	3.59 (1.86-6.95)	2.94 (1.50-5.79)
OCB	2.03 (1.19-3.47)	0.97 (0.54-1.72)	0.86 (0.47-1.56)
Dependencies	1.93 (1.06-3.51)	1.28 (0.68-2.40)	1.09 (0.56-2.09)

Model 1 = Odds ratio adjusted for age, sex, number of family members, and social class.
Model 2 = Odds ratio adjusted for age, sex, numbers of family members, social class, and all psychiatric factors.
Model 3 = Odds ratio adjusted for age, sex, numbers of family members, social class, all psychiatric factors, and history of psychiatric treatment.
